# No difference in anti-spike antibody and surrogate viral neutralization following SARS-CoV-2 booster vaccination in persons with HIV compared to controls (CO-HIV Study)

**DOI:** 10.3389/fimmu.2022.1048776

**Published:** 2023-01-09

**Authors:** Kendall D. Kling, Patrick Janulis, Alexis R. Demonbreun, Amelia Sancilio, Baiba Berzins, Karen Krueger, Chad Achenbach, Rachelle Price, Margaret Sullivan, Matthew Caputo, Sara Hockney, Teresa Zembower, Thomas W. McDade, Babafemi Taiwo

**Affiliations:** ^1^ Department of Medicine, Division of Infectious Diseases, Northwestern University Feinberg School of Medicine, Chicago, IL, United States; ^2^ Department of Pathology, Microbiology, Northwestern University Feinberg School of Medicine, Chicago, IL, United States; ^3^ Department of Medical Social Sciences, Northwestern University Feinberg School of Medicine, Chicago, IL, United States; ^4^ Department of Pharmacology, Northwestern University Feinberg School of Medicine, Chicago, IL, United States; ^5^ Institute for Policy Research, Northwestern University, Evanston, IL, United States; ^6^ Havey Institute for Global Health, Northwestern University Feinberg School of Medicine, Chicago, IL, United States; ^7^ Department of Anthropology, Northwestern University, Evanston, IL, United States

**Keywords:** SARS-CoV-2 antibody, neutralization, SARS-COV-2 vaccination, HIV, booster vaccines

## Abstract

**Background:**

Understanding the immune response to severe acute respiratory syndrome coronavirus 2 (SARS-CoV-2) vaccination will enable accurate counseling and inform evolving vaccination strategies. Little is known about antibody response following booster vaccination in people living with HIV (PLWH).

**Methods:**

We enrolled SARS-CoV-2 vaccinated PLWH and controls without HIV in similar proportions based on age and comorbidities. Participants completed surveys on prior SARS-CoV-2 infection, vaccination, and comorbidities, and provided self-collected dried blood spots (DBS). Quantitative anti-spike IgG and surrogate viral neutralization assays targeted wild-type (WT), Delta, and Omicron variants. We also measured quantitative anti-nucleocapsid IgG. The analysis population had received full SARS-CoV-2 vaccination plus one booster dose. Bivariate analyses for continuous outcomes utilized Wilcoxon tests and multivariate analysis used linear models.

**Results:**

The analysis population comprised 140 PLWH and 75 controls with median age 58 and 55 years, males 95% and 43%, and DBS collection on 112 and 109 days after the last booster dose, respectively. Median CD4 count among PLWH was 760 cells/mm^3^ and 91% had an undetectable HIV-1 viral load. Considering WT, Delta, and Omicron variants, there was no significant difference in mean quantitative anti-spike IgG between PLWH (3.3, 2.9, 1.8) and controls (3.3, 2.9, 1.8), respectively (*p*-values=0. 771, 0.920, 0.708). Surrogate viral neutralization responses were similar in PLWH (1.0, 0.9, and 0.4) and controls (1.0, 0.9, 0.5), respectively (*p*-values=0.594, 0.436, 0.706).

**Conclusions:**

PLWH whose CD4 counts are well preserved and persons without HIV have similar anti-spike IgG antibody levels and viral neutralization responses after a single SARS-CoV-2 booster vaccination.

## Introduction

Severe acute respiratory syndrome coronavirus 2 (SARS-CoV-2) emerged in late 2019 in China and subsequently spread to the rest of the world, including the United States (US) that has now seen over 88 million cases and over one million deaths (1). Mathematical modeling estimated that vaccination in the first year of its availability (December 2020 to December 2021) prevented 14.4 million deaths globally (2). The United States Food and Drug Administration (FDA) has approved four SARS-CoV-2 vaccines, namely the mRNA BNT162b2 and mRNA-1273 vaccines (manufactured by Pfizer-BioNTech and Moderna, respectively), an adenovirus vector vaccine Ad26.COV2.S (Johnson and Johnson), and NVX-CoV2373, a nanoparticle spike protein with adjuvant vaccine (Novavax). In-depth understanding of the immune response will enable accurate counseling and inform evolving recommendations on optimal vaccination schedule.

Despite 95% of the adult US population having any level of immunity to SARS-CoV-2 through vaccination or infection as of December 2021 (per the Centers for Disease Control and Prevention (CDC) seroprevalence data *via* blood donations), new infections continue to occur, even among previously infected, fully vaccinated, and boosted individuals (3). Reasons for breakthrough infections include inadequate immune protection following vaccination, waning antibody levels over time, and evasion of vaccine-induced immunity by new viral variants (4, 5). The concerns about breakthrough SARS-CoV-2 infection post-vaccination are heightened in immune compromised individuals, such as persons living with HIV (PLWH). Previous studies have suggested that PLWH produce less robust antibody in response to influenza (6) and hepatitis B (7) vaccination.

Several studies have evaluated antibody response to SARS-CoV-2 vaccination in PLWH, but few have included an adequate sample size, provided comparative data to the general population, or followed participants to determine durability. Overall, the results have been mixed, leaving many questions unanswered. One of the important unresolved questions is the robustness of antibody response after booster vaccination in PLWH, although one small study reported that PLWH had more variable and significantly lower anti-spike quantitative IgG levels less than 30 days after booster vaccination (8). To answer this question over a longer time period and in a larger sample, we designed this cross-sectional study comparing quantitative antibody response to SARS-CoV-2 spike protein and viral neutralization of the spike-ACE 2 protein post SARS-CoV-2 vaccination in PLWH versus controls without HIV. As a secondary objective, we measured quantitative nucleocapsid IgG to explore serologic evidence of prior infection in comparison to known SARS-CoV-2 infection in the cohort.

## Materials and methods

### Study population

The COVID-19 vaccination in HIV (CO-HIV) study was approved by the Institutional Review Board (IRB) at Northwestern University (STU00215691). Participants were identified between November 2021 and April 2022 from a Northwestern Medicine database of PLWH. We enrolled PLWH who had a record of SARS-CoV-2 vaccination about 6 months prior. A control group of participants without HIV was recruited from a registry of people from the general population who had expressed interest in participating in SARS-CoV-2 vaccine research. Controls were enrolled in similar proportions to the HIV group and matched based on six strata according to age (18-39, 40-59, and 60 and older) and presence or absence of any comorbidity in each age group. All participants were fully vaccinated (two doses of mRNA vaccines or one dose of adenovirus vector vaccine). Comorbidities were defined as diabetes, obesity, hypertension, asthma, heart disease, chronic lung disease, and kidney disease. Exclusion criteria included age less than 18 and any immunocompromising condition other than HIV (such as organ or stem cell transplantation, active chemotherapy, or current use of immunocompromising medication). Persons who reported prior SARS-CoV-2 infection were not excluded.

### Data collection

Eligible individuals were invited to participate by email and then contacted by telephone to provide informed consent electronically. Participants completed an initial online survey to report dates and types of SARS-CoV-2 vaccination (including any boosters), history of SARS-CoV-2 infection (including date, symptoms, how diagnosed, and if hospitalized), presence of any comorbidities, and pregnancy status. As PLWH were recruited through Northwestern Medicine, their self-reported vaccination dates were checked against the electronic medical records and corrected. Participants were considered to have prior infection if they self-reported a history, regardless of diagnostic testing information.

Consented participants were mailed a kit for self-collection of dried blood spots (DBS) – five drops of whole capillary blood self-collected by finger stick on filter paper (Whatman 903 Protein Saver Card). DBS samples were returned by pre-paid mail and stored at -20 degrees Celsius prior to analysis. Participants who returned DBS cards completed a follow-up survey to report any booster vaccination or SARS-CoV-2 diagnosis after the initial survey.

### Laboratory methods

Collected DBS samples were assayed in a batch at the Laboratory for Human Biology Research within the Anthropology department of Northwestern University in Evanston, Illinois. Quantitative anti-spike IgG (for wild type (WT), Delta, and Omicron variants), quantitative anti-nucleocapsid IgG, and surrogate viral neutralization based on inhibition of spike-ACE2 interaction were determined *via* electrochemiluminescent immunoassays on the Meso Scale Diagnostics platform. These assays have shown high sensitivity and specificity compared with matched serum samples (9–12).

For the anti-spike and anti-nucleocapsid assays, DBS samples first were punched into discs *via* pneumatic device (Analytic Sales and Services #327,500, Flanders, NJ) then added to a dipotassium phosphate-buffered saline with 0.5% sodium azide and 1.5% bovine serum albumin. The eluate was then diluted and then the testing was performed *via* the Meso Scale Discovery multiplex anti-IgG chemiluminescence assay (SARS-CoV-2 Panel 24 K15575U). We obtained quantitative anti-spike IgG against Wild-type (Wuhan A), Delta (B.1.617.2;AY.2), and Omicron (B.1.1.529; BA.1). Ranges for the anti-spike IgG for WT, Delta, and Omicron were 0.0175-70 AU/mL, 0.009-40 AU/mL, and 0.001-6 AU/mL, respectively. Ranges for anti-nucleocapsid IgG were 0.0170-70 AU/mL. For anti-nucleocapsid IgG response, we defined a cutoff of greater than 0.19 AU/mL, which is three standard deviations above the mean of pre-pandemic negative samples, as a proxy for prior SARS-CoV-2 infection (13). The Meso Scale Discovery antibody assay (Lot Number K0081945) conversion from MSD units (AU/mL) to WHO/NIBSC units (BAU/mL) can be calculated by multiplying MSD units by a conversion factor (0.00901 for anti-spike IgG and 0.00236 for anti-nucleocapsid IgG) (14).

For the surrogate viral neutralization assay, DBS samples were initially hole punched with a pneumatic device (Analytic Sales and Services #327,500, Flanders, NJ). The 5 mm discs created were eluted overnight in assay diluent, then transferred to solid phase plate coated with spike antigen of SARS-CoV-2. Recombinant ACE-2 bound to electrochemiluminescent label (K15386U-2, Meso Scale Diagnostics) was then added to the plate, then washed, followed by addition of read buffer. Inhibition of binding between ACE2 and spike protein was detected *via* mean fluorescence intensity (MFI). Calculation of percent neutralization performed as 100 x 1 – (sample MFI/negative control MFI).

### Statistical analyses

The CDC updated the recommendation for SARS-CoV-2 vaccination while this study was being planned and implemented (15); hence, most study participants had received a booster dose by the time of the first DBS sample collection, while few had received more than one booster dose or none. The analysis population was restricted to participants who reported a single booster dose (n=215). We examined differences in log transformed values of IgG response to spike amongst different variants (WT, Delta, and Omicron) and IgG to nucleocapsid comparing PLWH to the general population. In addition, we also examined neutralization response across each variant again comparing PLWH to controls. Bivariate analyses for continuous outcomes utilized Wilcoxon rank sum unpaired test (i.e., Mann-Whitney U test) and multivariate analysis used generalized linear models with a gaussian distribution. Multivariate models include the following covariates: primary series vaccine type, days since last vaccine (coded: 1-2 months, 2-4 months, 4-6 months, or 6-8 months), report of prior SARS-CoV-2 diagnoses, comorbidities (i.e., diabetes, hypertension, asthma, heart disease, obesity, or other comorbidity), age (coded: <40, 40-59, ≥60), and sex, except for models examining differences in reported infection and asymptomatic infection where SARS-CoV-2 diagnoses was not included as a covariate. Asymptomatic infection was defined as serologic evidence of prior infection in a person who indicated on the survey that they had never been infected.

## Results

We consented and mailed DBS kits to 317 participants, and 77% (243) returned DBS samples (166 PLWH and 77 controls) (**Supplemental Table**). The analysis population comprised 215 participants (140 PLWH and 75 controls) who received full vaccination plus one booster dose prior to DBS sample collection (**Table 1**). PLWH and controls were comparable in age (mean 58 versus 55 years), while sex was imbalanced (95% male in PLWH compared to 43% male in controls without HIV). The groups were similar in type of vaccine received, time between booster dose and sample collection (mean of 112 days in PLWH versus 109 days in controls). PLWH had a median CD4+ T lymphocyte (CD4) count of 760 cells/mm^3^.

**Table 1:** Participant characteristics (analysis group).

Variable	PLWH n (%)	Controls n (%)
Total	140 (100.0)	75 (100.0)
Age*	58.0 (10.1)	54.9 (12.4)
Age Group		
< 40	7 (5.0)	9 (12.0)
40 – 59	64 (45.7)	34 (45.3)
≥ 60	69 (49.3)	32 (42.6)
Sex		
Female	7 (5.0)	43 (57.3)
Male	133 (95.0)	32 (42.7)
Race		
Asian	4 (2.9)	3 (4.0)
Black or African American	9 (6.4)	0 (0.0)
Native Hawaiian or other Pacific Islander	0 (0.0)	1 (1.3)
Other	4 (2.9)	1 (1.3)
White	120 (85.7)	62 (82.7)
Missing/Declined	3 (2.1)	8 (10.7)
Ethnicity		
Hispanic	12 (8.6)	1 (1.3)
Non-Hispanic	126 (90.0)	66 (88.0)
Missing/Declined	2 (1.4)	8 (10.7)
Comorbidities: Any	78 (55.7)	36 (48)
Diabetes	15 (10.7)	5 (6.7)
Hypertension	49 (35.0)	16 (21.3)
Asthma	13 (9.3)	12 (16.0)
Heart disease	14 (10.0)	3 (4.0)
Obesity	13 (9.3)	16 (21.3)
Chronic lung diseases (e.g., COPD)	2 (1.4)	0 (0.0)
Kidney disease	6 (4.2)	0 (0.0)
Vaccine Type (Primary)		
Pfizer (BNT162b2)	94 (67.1)	54 (72.0)
Moderna (mRNA-1273)	45 (32.1)	19 (25.3)
J&J (Ad26.COV2.S)	1 (0.7)	2 (2.7)
Days since 1^st^ booster**	111.5 (45-153.25)	109 (83.5 - 128)
Self-reported history of SARS-CoV-2 infection		
No	118 (84.2)	60 (78.7)
Yes	21 (15.0)	16 (21.3)
CD4 (cells/mm^3^) *	760.2 (93 – 1639)	–
Viral Load (HIV-1 RNA, copies/mL)		
< 20 (Undetectable)	128 (91.4)	–
20 to 500	10 (7.1)	–
> 500	2 (1.4)	–

*Values are mean (standard deviation). ** Values are median (IQR).

**Figure 1:**
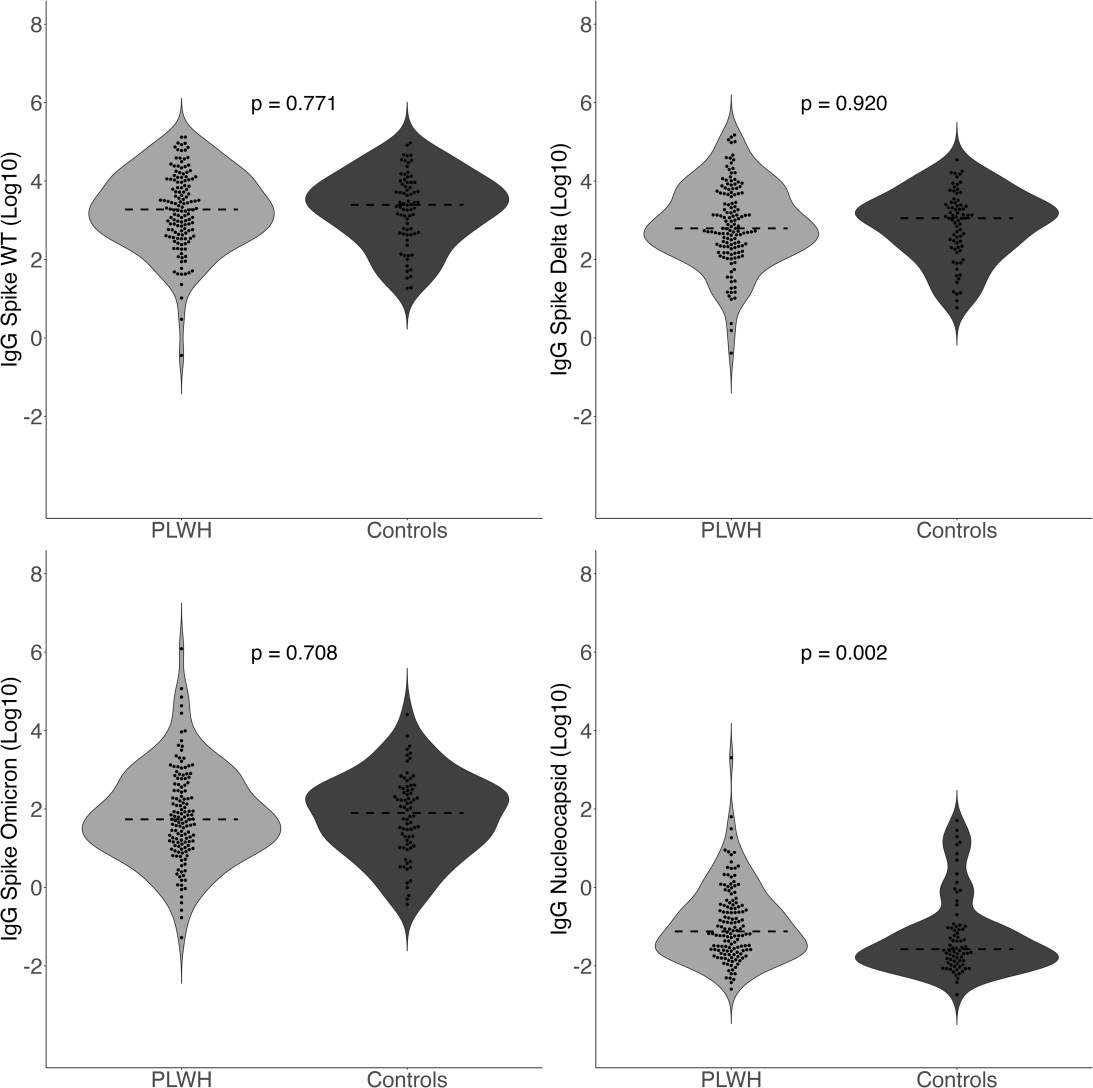
Quantitative IgG Response to spike amongst different variants (WT, Delta, Omicron) and IgG to nucleocapsid comparing PLWH to controls. Dashed line indicates the group median value.

There was no significant difference in anti-spike IgG values between PLWH and controls without HIV across any of the variants (**Figure 1**). The mean anti-spike IgG in AU/mL to WT was 3.3 [95% CI: 3.1, 3.5] log_10_ for PLWH and 3.3 [95% CI: 3.1, 3.6] log_10_ for controls (*p*-value= 0.771). The mean value of IgG to spike Delta was 2.9 [95% CI: 2.7, 3.1] log_10_ for PLWH and 2.9 [95% CI: 2.7, 3.1] log_10_ for controls (*p*-value= 0.920). The mean IgG level to spike Omicron was 1.8 [95% CI: 1.6, 2.0] log_10_ for PLWH and 1.8 [95% CI: 1.6, 2.1] log_10_ for controls (*p*-value= 0.708).

**Figure 2:**
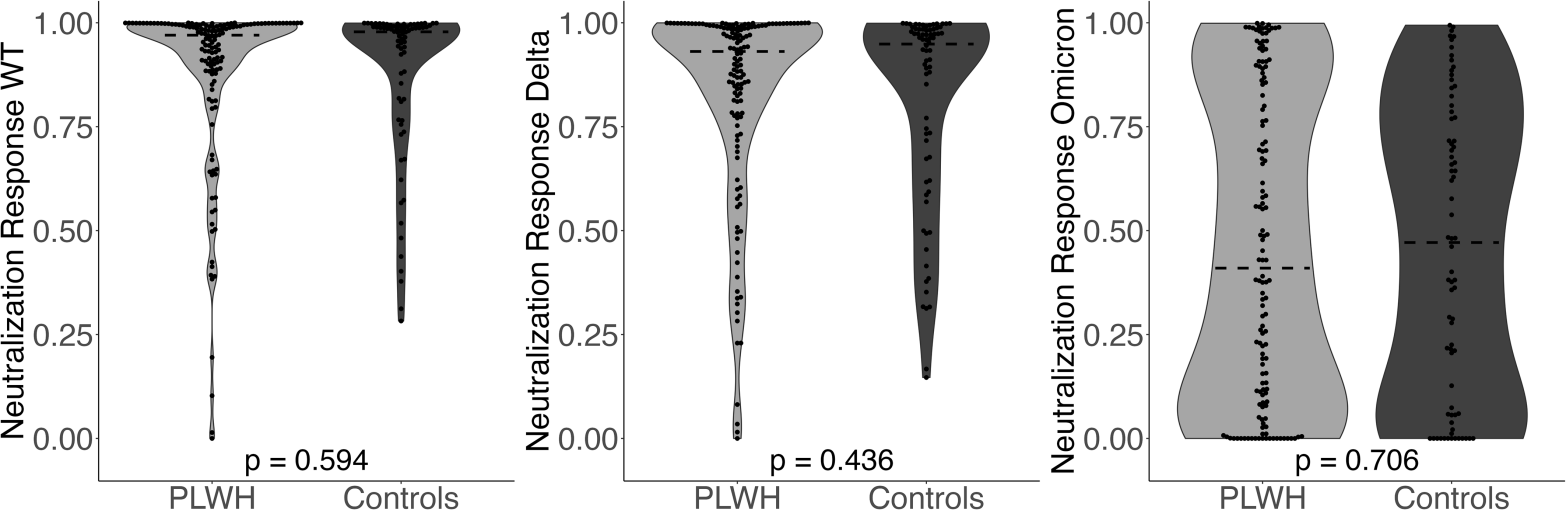
Neutralization Response (WT, Delta, Omicron) comparing PLWH to controls. Dashed line indicates the group median value.

For percent neutralization, there were no significant differences across PLWH and controls without HIV for any variants (**Figure 2**). The median value for neutralization response against WT was 1.0 [IQR 0.9, 1.0] log_10_ for PLWH and 1.0 [IQR 0.8, 1.0] log_10_ for controls (*p*-value=0.594). The median value for neutralization response against Delta was 0.9 [IQR 0.8, 1.0] log_10_ for PLWH and 0.9 [IQR 0.7, 1.0] log_10_ for controls (*p*-value=0.436). The median value for neutralization response against Omicron was 0.4 [IQR 0.1, 0.9] log_10_ for PLWH and 0.5 [IQR 0.1, 0.8] log_10_ for controls (*p*-value=0.706). Again, the results of the analysis did not change after controlling for covariates in multivariate models.

Anti-nucleocapsid IgG concentrations in AU/mL were significantly higher among PLWH (*p*-value= 0.002) with a median value of -1.1 [IQR -1.6, -0.4] log_10_ for PLWH and -1.6 [IQR -1.9, -1.0] log_10_ for controls without HIV (*p*-value= 0.002). Multivariate results were identical to those in the bivariate analyses. PLWH (77.9%; 109/140) were more likely than controls (56.8%; 42/74) to have anti-nucleocapsid IgG greater than 0.19 AU/mL (χ^2^ = 9.39, *p*-value= 0.002), indicating serologic evidence of prior infection. Regarding prior self-reported history of SARS-CoV-2 infection, there was no significant difference between PLWH (15.0%; 21/140) and controls (21.3%; 16/75; χ^2^ = 0.96, *p*-value= 0.326). As such, protocol-defined asymptomatic infection was more common in PLWH (63.6%; 89/140) than controls (37.3%; 28/75), (χ^2^ = 12.52, *p*-value= < 0.001). Multivariate results were identical to those in the bivariate analyses.

Finally, we conducted two types of sensitivity analysis. First, we conducted a series of stratified bivariate analyses for male only, by age (i.e. less than 40, 40-59, and 60 and older), and by time since booster (i.e., 1-2 months 3-4 months, 4-5 months, and 6-8 months). We used Wilcoxon rank sum unpaired test and adjusting for multiple comparisons using the Benjamini-Hochberg procedure (16). In these analyses, we found no significant differences between PLWH and controls (**Supplemental Figures 1**-**16**). Second, to correct for potential error in self-report of prior SARS-CoV-2 infection without laboratory confirmation in multivariate models, we conducted a sensitivity analysis controlling for laboratory confirmed infection (i.e., *via* nasal swab or antibody test) and found results identical to our main multivariate and bivariate analyses.

## Discussion

Vaccination reduces occurrence of severe disease, hospitalizations, and deaths from SARS-CoV-2 and is particularly important for populations at higher risk of adverse outcomes, such as PLWH (17). In this study, we found that quantitative anti-spike IgG and neutralization values at a median of 112 days after a booster dose in PLWH with preserved CD4 counts were similar to controls without HIV. The finding was consistent across the WT, Delta, and Omicron variants tested. As expected, anti-spike IgG titers and viral neutralization responses were lowest against Omicron variant which has been shown to evade much of the protection from vaccination (18).

Only one published study has evaluated antibody response post-booster vaccination in PLWH to our knowledge (8). In the study, anti-spike IgG, anti-spike IgA, anti-nucleocapsid IgG, and neutralization response against SARS-CoV-2 were measured in PLWH and controls who had received the BNT162b2 vaccine. In the PLWH group (median CD4 count 577 cells/mm^3^), 52 participants were tested at a mean of 26 days post-booster compared to 41 health workers without HIV infection. Findings of the study contrasted ours in that they found PLWH had variable and significantly lower anti-spike quantitative IgG levels than controls. Potential reasons for this difference could be that our cohort of PLWH was larger (140 versus 52) with a longer mean time to sample collection after the booster (105 days versus 26 days) and on average higher CD4 count (median 760 versus 577 cells/mm^3^).

Consistent with our results, other studies have found comparable quantitative anti-spike IgG and viral neutralization responses following SARS-CoV-2 vaccination in immune competent PLWH and persons without HIV, but did not report boosting data. One was a prospective cohort study of 71 PLWH who had received two doses of the mRNA-1273 vaccine (19). The investigators collected samples before the first dose, 28 days after the first dose, and 28 days after the second dose, and found no statistically significant difference in anti-spike IgG and viral neutralization response when compared to ten volunteers without HIV infection (19). Another group of investigators compared 100 PLWH to 152 controls and measured anti-spike antibody levels, viral neutralization, and angiotensin converting enzyme 2 (ACE2) displacement at various intervals after vaccination (one month after the first dose, one month after the second dose, and three months after the second dose) (20). They found that PLWH had lower antibody and ACE2 displacement after the first dose of the vaccine, but this was not seen after the second dose (20).

On the other hand, some studies have reported lower immune response after full vaccination among PLWH compared to those without HIV, also without evaluating post-boosting effects. In the study of 100 vaccinated PLWH (excluded natural infection) and 100 matched controls without HIV, there was 2.4-fold greater odds of pseudovirus neutralization antibody non-response among PLWH in comparison to people without HIV (21). Interestingly, the study also found that low CD4 T cell count and unsuppressed HIV-1 plasma viral load were some of the factors associated with lower overall neutralizing antibody titers (21). The lower immune response among PLWH in the study could have been related to the CD4 count of the cohort, which was a median of 511 cells/mm^3^ in contrast to a median of 760 in our study. Indeed, a study of 105 PLWH found that when CD4 counts were less than 500 cells/mm^3^, and especially less than 200 cells/mm^3^, quantitative SARS-CoV-2 anti-spike IgG titers tended to be less robust after vaccination (22). Another study analyzed quantitative SARS-CoV-2 anti-spike IgG in 121 PLWH compared to 20 controls 3-4 weeks after receiving one dose of SARS-CoV-2 mRNA vaccine and found a lower antibody response only when CD4 counts were less than 250 cells/mm^3^ (23).

Another study also reported consistently lower antibody titer level post-vaccination for PLWH at multiple time points; however, the median age of PLWH in the study was 54 years compared to 30 years in the comparators (24). Their results could have been affected by age imbalance between the groups since evidence suggests older individuals have lower quantitative anti-spike IgG after vaccination (25).

As an exploratory objective, we quantified anti-nucleocapsid IgG, using levels above 0.19 AU/mL as a proxy for prior infection (13). One interesting finding was that more PLWH had anti-nucleocapsid IgG levels that were indicative of prior infection, despite reporting similar rates of symptomatic SARS-CoV-2 infection as controls without HIV. This observation could indicate that asymptomatic SARS-CoV-2 infection may be more common in PLWH, but it should be interpreted with caution. A limitation to this finding is that participants were considered to have a history of SARS-CoV-2 infection based on survey response; however, our results were confirmed in sensitivity analysis that focused on those with laboratory confirmed infection. Further, anti-nucleocapsid IgG levels tend to wane over time with approximately 39% of people with documented infection having undetectable levels by 16 weeks after infection, while other persons may not develop anti-nucleocapsid antibodies (26). Recent evidence also suggests that anti-spike vaccination can induce production of anti-nucleocapsid antibodies, further confounding the results (27). Nevertheless, our observation of possibly higher occurrence of asymptomatic SARS-CoV-2 infection in PLWH deserves further study.

The results in this study may not be generalizable to all PLWH as 91% of the PLWH in our study had an HIV-1 viral load that was undetectable, 94% had a CD4 count > 350 cells/mm^3^, and 44% reported no comorbidities. Results may be different in PLWH with uncontrolled viremia, CD4 counts below 200 cells/mm^3^, or multiple comorbidities. Another limitation is a striking sex imbalance between the groups (95% male in PLWH compared to 43% in the control group). The imbalance occurred because PLWH were recruited from our clinic population which is predominantly male while the control group was recruited from a registry composed of the general population. Moreover, our recruitment strategy targeted a balance in age and comorbidities, but not sex. It is unlikely that the sex imbalance significantly affected our conclusions. In fact, our results could have been biased towards a lower antibody response in the PLWH group since some data suggests that women produce higher levels of anti-spike IgG than men after SARS-CoV-2 vaccination (28).

Guidelines for SARS-CoV-2 vaccination are still evolving with advances in knowledge; a second booster vaccine dose is now recommended after full vaccination in high-risk individuals (14). In-depth understanding of the immune response following booster vaccination is critical for accurate patient counseling and to inform vaccine strategies. Overall, this study provides reassurance that PLWH with preserved immune competence (based on high CD4 cell counts) have comparable humoral immune response following SARS-CoV-2 booster vaccination as the general population, at least in the short-term. This information is empowering to PLWH and helpful to clinicians counseling them on the benefits of SARS-CoV-2 vaccination. The PLWH and persons without HIV enrolled in this study continue to be followed to compare trends in anti-spike antibody levels and viral neutralization response over time.

## Data availability statement

The raw data supporting the conclusions of this article will be made available by the authors, without undue reservation.

## Ethics statement

The studies involving human participants were reviewed and approved by This study was approved by the Institutional Review Board (IRB) at Northwestern University (STU00215691). The patients/participants provided their written informed consent to participate in this study.

## Author contributions

KK formulated the project and composed the majority of the manuscript. PJ performed the data and statistical analysis and generated the figures and tables. AD and AS developed and performed the laboratory assays. BB helped formulate the structure of the project and was critical to organizing our database. KK helped devise the plan for the study and helped to consent participants. RP, SH, and MS contributed to literature review and consent of participants. MC assisted with data retrieval from our data sets. CA and TZ provided expert consultation on laboratory assays and study design. TM oversaw the laboratory that performed the assays and helped develop the tests. BT is the PI of the project and was instrumental to the genesis of the project. All authors contributed to the article and approved the submitted version.

## Supplementary material

Supplementary Figure 1 Quantitative IgG Response to spike amongst different variants (WT, omicron, delta) and IgG to nucleocapsid comparing PLWH to controls (males only). Dashed line indicates the group median value.

Supplementary Figure 2 Neutralization Response (WT, delta, omicron) comparing PLWH to controls (males only). Dashed line indicates the group median value.

Supplementary Figure 3 Quantitative IgG Response to spike amongst different variants (WT, omicron, delta) and IgG to nucleocapsid comparing PLWH to controls (age less than 40 only). Dashed line indicates the group median value.

Supplementary Figure 4 Neutralization Response (WT, delta, omicron) comparing PLWH to controls (age less than 40 only). Dashed line indicates the group median value.

Supplementary Figure 5 Quantitative IgG Response to spike amongst different variants (WT, omicron, delta) and IgG to nucleocapsid comparing PLWH to controls (age 40-60 only). Dashed line indicates the group median value.

Supplementary Figure 6 Neutralization Response (WT, delta, omicron) comparing PLWH to controls (age 40-60 only). Dashed line indicates the group median value.

Supplementary Figure 7 Quantitative IgG Response to spike amongst different variants (WT, omicron, delta) and IgG to nucleocapsid comparing PLWH to controls (age over 60 only). Dashed line indicates the group median value.

Supplementary Figure 8 Neutralization Response (WT, delta, omicron) comparing PLWH to controls (age over 60 only). Dashed line indicates the group median value.

Supplementary Figure 9 Quantitative IgG Response to spike amongst different variants (WT, omicron, delta) and IgG to nucleocapsid comparing PLWH to controls (less than 2 months since last vaccine dose only). Dashed line indicates the group median value.

Supplementary Figure 10 Neutralization Response (WT, delta, omicron) comparing PLWH to controls (less than 2 months since last vaccine dose only). Dashed line indicates the group median value.

Supplementary Figure 11 Quantitative IgG Response to spike amongst different variants (WT, omicron, delta) and IgG to nucleocapsid comparing PLWH to controls (2-4 months since last vaccine only). Dashed line indicates the group median value.

Supplementary Figure 12 Neutralization Response (WT, delta, omicron) comparing PLWH to controls (2-4 months since last vaccine only). Dashed line indicates the group median value.

Supplementary Figure 13 Quantitative IgG Response to spike amongst different variants (WT, omicron, delta) and IgG to nucleocapsid comparing PLWH to controls (4-6 months since last vaccine only). Dashed line indicates the group median value.

Supplementary Figure 14 Neutralization Response (WT, delta, omicron) comparing PLWH to controls (4-6 months since last vaccine only). Dashed line indicates the group median value.

Supplementary Figure 15 Quantitative IgG Response to spike amongst different variants (WT, omicron, delta) and IgG to nucleocapsid comparing PLWH to controls (6-8 months since last vaccine only). Dashed line indicates the group median value.

Supplementary Figure 16 Neutralization Response (WT, delta, omicron) comparing PLWH to controls (6-8 months since last vaccine only). Dashed line indicates the group median value.
