# Supplementary figures and images for: No difference in anti-spike antibody and surrogate viral neutralization following SARS-CoV-2 booster vaccination in persons with HIV compared to controls (CO-HIV Study)
Source: Front Immunol. 2023 Jan 9;13:1048776. doi: 10.3389/fimmu.2022.1048776 (PMC9868861; doi:10.3389/fimmu.2022.1048776)

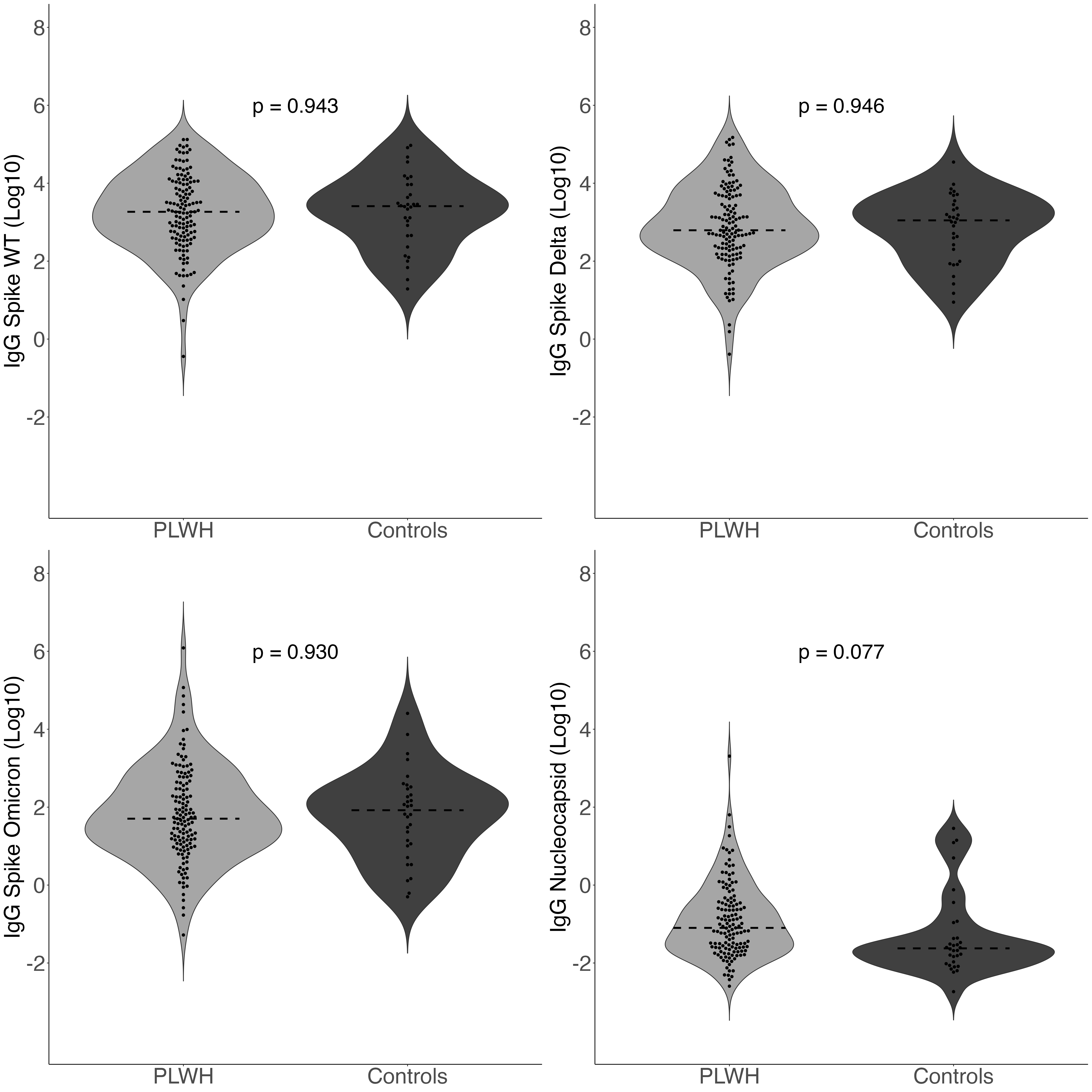

Supplement: Supplementary Figure 1 — Quantitative IgG Response to spike amongst different variants (WT, omicron, delta) and IgG to nucleocapsid comparing PLWH to controls (males only). Dashed line indicates the group median value. [file DataSheet_1.zip › COHIVSupplementalFigure1.tif]
