# Supplementary material for: No difference in anti-spike antibody and surrogate viral neutralization following SARS-CoV-2 booster vaccination in persons with HIV compared to controls (CO-HIV Study)
Source: Front Immunol. 2023 Jan 9;13:1048776. doi: 10.3389/fimmu.2022.1048776 (PMC9868861; doi:10.3389/fimmu.2022.1048776)
Supplement: Supplementary Figure 1 — Quantitative IgG Response to spike amongst different variants (WT, omicron, delta) and IgG to nucleocapsid comparing PLWH to controls (males only). Dashed line indicates the group median value. [file DataSheet_1.zip › COHIVSupplementalTable.zip!/COHIVSupplementalTable.docx]

**Supplemental Table.** All participant characteristics.

| **Variable** | **PLWH**  n (%) | **Controls**  n (%) |
| --- | --- | --- |
| Total | 166 (100.0) | 77 (100.0) |
| Age* | 57.4 (25 – 81) | 55.1 (28 – 77) |
| Sex |  |  |
| Female | 11 (6.6) | 43 (55.8) |
| Male | 155 (32.4) | 34 (44.2) |
| Race |  |  |
| Asian | 4 (2.4) | 3 (3.9) |
| Black or African American | 12 (7.2) | 1 (1.3) |
| Native Hawaiian or other Pacific Islander | 0 (0.0) | 1 (1.3) |
| Other | 5 (3.0) | 1 (1.3) |
| White | 141 (84.9) | 63 (81.8) |
| Missing/Declined | 4 (2.4) | 8 (10.4) |
| Ethnicity |  |  |
| Hispanic | 15 (9.0) | 1 (1.3) |
| Non-Hispanic | 149 (89.8) | 68 (88.3) |
| Missing/Declined | 2 (1.2) | 8 (10.4) |
|  |  |  |
| Comorbidities: Any | 94 (56.6) | 37 (48.1) |
| Diabetes | 18 (10.8) | 5 (6.4) |
| Hypertension | 58 (34.9) | 17 (22.1) |
| Asthma | 18 (10.8) | 12 (15.6) |
| Heart disease | 17 (10.2) | 3 (3.9) |
| Obesity | 16 (9.6) | 16 (20.8) |
| Chronic lung diseases (e.g., COPD) | 4 (2.4) | 0 (0.0) |
| Kidney disease | 8 (4.8) | 0 (0.0) |
|  |  |  |
| Vaccine Type (Primary) |  |  |
| Pfizer (BNT162b2) | 113 (68.0) | 56 (72.7) |
| Moderna (mRNA-1273) | 52 (31.3) | 19 (24.7) |
| J&J (Ad26.COV2.S) | 1 (0.6) | 2 (2.6) |
|  |  |  |
| Days since 1^st^ booster* | 115.7 (2 – 338) | 113.9 (20 - 351) |
|  |  |  |
| Self-reported history of SARS-CoV-2 infection |  |  |
| No | 141 (84.9) | 61 (79.2) |
| Yes | 24 (14.5) | 16 (20.8) |
| Missing | 1 (0.6) | 0 (0.0) |
|  |  |  |
| CD4 (cells/mm^3^)* | 739.4 (93 – 1682) | - |
| Viral Load (HIV-1 RNA, copies/mL) |  |  |
| < 20 | 151 (91.0) | - |
| 20 to 500 | 9 (5.4) | - |
| > 500 | 6 (3.6) | - |

*^Values are mean (range).^
